# Life‐cycle and cost of goods assessment of fed‐batch and perfusion‐based manufacturing processes for mAbs

**DOI:** 10.1002/btpr.2323

**Published:** 2016-07-28

**Authors:** Phumthep Bunnak, Richard Allmendinger, Sri V. Ramasamy, Paola Lettieri, Nigel J. Titchener‐Hooker

**Affiliations:** ^1^ Dept. of Biochemical EngineeringUniversity College LondonLondonU.K.; ^2^ Alliance Manchester Business SchoolUniversity of ManchesterManchesterU.K.; ^3^ Dept. of Chemical EngineeringUniversity College LondonLondonU.K.

**Keywords:** life‐cycle assessment (LCA), monoclonal antibody, environmental assessment, sustainability, decision‐making

## Abstract

Life‐cycle assessment (LCA) is an environmental assessment tool that quantifies the environmental impact associated with a product or a process (e.g., water consumption, energy requirements, and solid waste generation). While LCA is a standard approach in many commercial industries, its application has not been exploited widely in the bioprocessing sector. To contribute toward the design of more cost‐efficient, robust and environmentally‐friendly manufacturing process for monoclonal antibodies (mAbs), a framework consisting of an LCA and economic analysis combined with a sensitivity analysis of manufacturing process parameters and a production scale‐up study is presented. The efficiency of the framework is demonstrated using a comparative study of the two most commonly used upstream configurations for mAb manufacture, namely fed‐batch (FB) and perfusion‐based processes. Results obtained by the framework are presented using a range of visualization tools, and indicate that a standard perfusion process (with a pooling duration of 4 days) has similar cost of goods than a FB process but a larger environmental footprint because it consumed 35% more water, demanded 17% more energy, and emitted 17% more CO_2_ than the FB process. Water consumption was the most important impact category, especially when scaling‐up the processes, as energy was required to produce process water and water‐for‐injection, while CO_2_ was emitted from energy generation. The sensitivity analysis revealed that the perfusion process can be made more environmentally‐friendly than the FB process if the pooling duration is extended to 8 days. © 2016 American Institute of Chemical Engineers *Biotechnol. Prog*., 32:1324–1335, 2016

## Introduction

The design of a manufacturing process for biopharmaceuticals, such as monoclonal antibodies (mAbs), or any other commercial product is based on various criteria, such as capital investment, operating costs, process reliability and safety, and environmental impact.^1^ While research on the economics of biomanufacturing processes has become popular in the last 10 years,^2^, ^3^, ^4^, ^5^, ^6^ there is little research and insight into the environmental impacts of adopting particular biomanufacturing processes and different biomanufacturing technologies. Life‐cycle assessment (LCA)^7^, ^8^ is a systematic method that focuses on describing the environmental consequences of each element in a process but has not been used widely within the bioprocessing sector. The goal of this work is to propose an LCA‐based framework to contribute toward designing more cost‐efficient, robust and environmentally‐friendly manufacturing processes for mAbs, which are arguably the highest selling class of biopharmaceuticals with a sales value of approximately $24.6 billion in 2012 in the United States.^9^

Currently, the large‐scale production of mAbs is based on production systems that use recombinant mammalian cells.^10^ *Fed‐batch (FB) bioreactors* have become the default platform technology for large‐scale production of mAb due to their ease of scalability (up to 20,000 L), robustness, and high volumetric productivity.^11^ In contrast to a FB bioreactor, a *perfusion bioreactor* operates continuously by feeding and withdrawing the culture media while retaining cells within the bioreactor.^12^, ^13^ The operability of a new piece of equipment or a process needs to be weighed against the resulting economic advantages and environmental burdens. While there is literature emerging on the economic impact of different bioreactor types,^3^, ^6^ the environmental burdens remain rather unexplored. To gain a better understanding of the trade‐offs between the economic and environmental impact of FB and perfusion‐based manufacturing processes, we present here an LCA‐based framework comprising an LCA modeling software (GaBi) and an industry standard bioprocess model (BioSolve). The framework has the ability to assess a specific manufacturing process as well as highlight the impact of uncertainties in process parameters and production scale‐up on economic and environmental metrics.

### Related research

Previous work on evaluating the environmental impact of biopharmaceutical manufacturing was not based on LCA but largely on two metrics, namely the process mass intensity (PMI) or the E‐factor.^3^, ^14^, ^15^ The PMI^15^ is calculated by dividing the total input (kg) of starting materials, reagents, solvents and process water by the output (kg) product, while the E‐factor^16^ is defined by the ratio of the mass of waste per unit of product. Traditionally, the PMI and E‐factor do not assess water consumption of non‐process related steps nor the cumulative energy demand.^17^ The American Chemical Society Green Chemistry Institute® (ACS GCI) Pharmaceutical Roundtable selected PMI to benchmark processes across the pharmaceutical industry.^15^ Ho et al.^14^ used the E‐factor to assess the impact of therapeutic biologics in general. Similar to this study, Pollock et al.^3^ compared FB and perfusion‐based processes using the E‐factor. Pollock et al. computed E‐factor values for the water consumption of process and non‐process related steps but did not evaluate aspects related to energy consumption and CO_2_ emissions, which contribute significantly to the overall environmental assessment of a process as will be demonstrated later.

Compared with the PMI and E‐factor, LCA is a more comprehensive environmental assessment tool^15^ and accounts for the environmental impact of the individual production stages ranging from raw material extraction to equipment disposal. Unlike the PMI and E‐factor, LCA allows various additional impact categories to be evaluated, such as toxicological impacts, global warming potential, acidification, and loss of biodiversity. Only a few LCA studies have been conducted to date with all focusing on the environmental assessment of a traditional fixed‐in‐place stainless‐steel facility versus a facility that relies on single‐use equipment.^18^, ^19^, ^20^, ^21^ Arguably, the first streamlined LCA study within the biopharma sector was conducted by GE Healthcare in collaboration with Yale University in 2009 based on a WAVE 500 single‐use bioreactor.^19^ Following this study, GE Healthcare initiated a major LCA study in collaboration with BioPharm Services Ltd and GE's Global Research Ecoassessment Center of Excellence.^20^ The goal of the study was to compare the use of single‐use versus traditional durable process technologies at levels of 100, 500, and 200 L scales. The results indicated that a single‐use process train exhibited lower environmental impact compared with the traditional fixed‐in‐place process train in each environmental impact category (17 in total) studied. This observation was primarily due to the reduced need for energy and water intensive process steps that are required for traditional fixed‐in‐place equipment. This study will show that, in the context of FB and perfusion‐based processes, there are certain conditions (e.g., as a function of the perfusion pool duration) at which “environmental friendliness” can switch from one process platform to another. Finally, the most recent work on LCA within the biopharmaceutical industry provides methodological guidelines on the application of LCA in the design of environmentally‐friendly biomanufacturing processes, and also proposes a decision‐support LCA tool to achieve this.^22^

The LCA‐based framework proposed in this study follows established guidelines,^22^ and derives process costs and facility data using models developed by Biopharm Services Ltd, BioSolve, which was already used in Ref. 20. The application of the framework to the environmental assessment of FB and perfusion‐based bioprocesses is complementary to previous work that focused solely on assessing single‐use and traditional multi‐use bioprocessing systems.

## Methodology

This first part of this section introduces briefly the steps that constitute LCA, and describes the system boundary considered in this work to validate the environmental impact of two commonly used mAb manufacturing technologies.

### Life‐cycle assessment

Applying the concept of life‐cycle thinking avoids shifting the environmental burden from one production phase to another. This is achieved through the implementation of four stages:^23^ First, the purpose of the study, the system boundary, and the type of information needed are defined in the *goal and scope definition stage*. This is followed by a *life‐cycle inventory (LCI) analysis*, where mass and energy balances across the system boundaries are quantified. Consequently, the data from the LCI analysis is used to evaluate the different environmental impacts within the *life‐cycle impact assessment*. Finally, the results are analyzed and necessary process modifications suggested in the *interpretation stage*. Arguably, while water consumption, largely related to clean‐in‐place (CIP) and steam‐in‐place (SIP) systems, represents a major environmental burden, biopharmaceutical manufacturing can impact the environment in various other ways too including (liquid and solid) waste generation, energy consumption, and greenhouse gas (GHG) emission.^24^ In particular, waste generation can pose a great environmental challenge as plastic waste is generally not recycled due to its multicomponent nature.^25^ Current options to dispose the waste include landfill, incineration, and pyrolysis.^26^ Simulating and understanding the impact of these options in the context of biopharmaceutical manufacturing is a major objective of this work.

Energy consumption is also associated with a large footprint in the biopharma sector due to the necessity to maintain identical ambient operating conditions so to ensure consistent product quality. In fact, the energy dedicated to a heating, ventilation, and air conditioning (HVAC) system is estimated to constitute 65% of the total energy requirement of a pharmaceutical plant.^51^ Other energy‐intense operations in a biopharmaceutical plant include the production of purified water (PW) and water‐for‐injection (WFI), equipment cleaning and sterilization, mass and heat transfer arising in upstream processes, and fluid transport and mixing present in downstream processes.^14^ An increasing awareness and interest of the industry in climate change is also now reflected in environmental concerns over GHG emission levels.^21^ Typical sources of GHG emissions in a biopharmaceutical plant include electricity and steam generation, fermentation, and transportation of facility workers.

While previous LCA studies compared the impact of single‐use versus multi‐use bioprocess systems,^18^, ^19^, ^20^, ^21^ this current study goes a step further and demonstrates how LCA can be linked to a bioprocess simulation model to select a manufacturing strategy based on either FB or perfusion bioreactors with the goal to minimize water consumption, levels of solid waste generation, energy requirement, and CO_2_ emissions.

**Figure 1:**
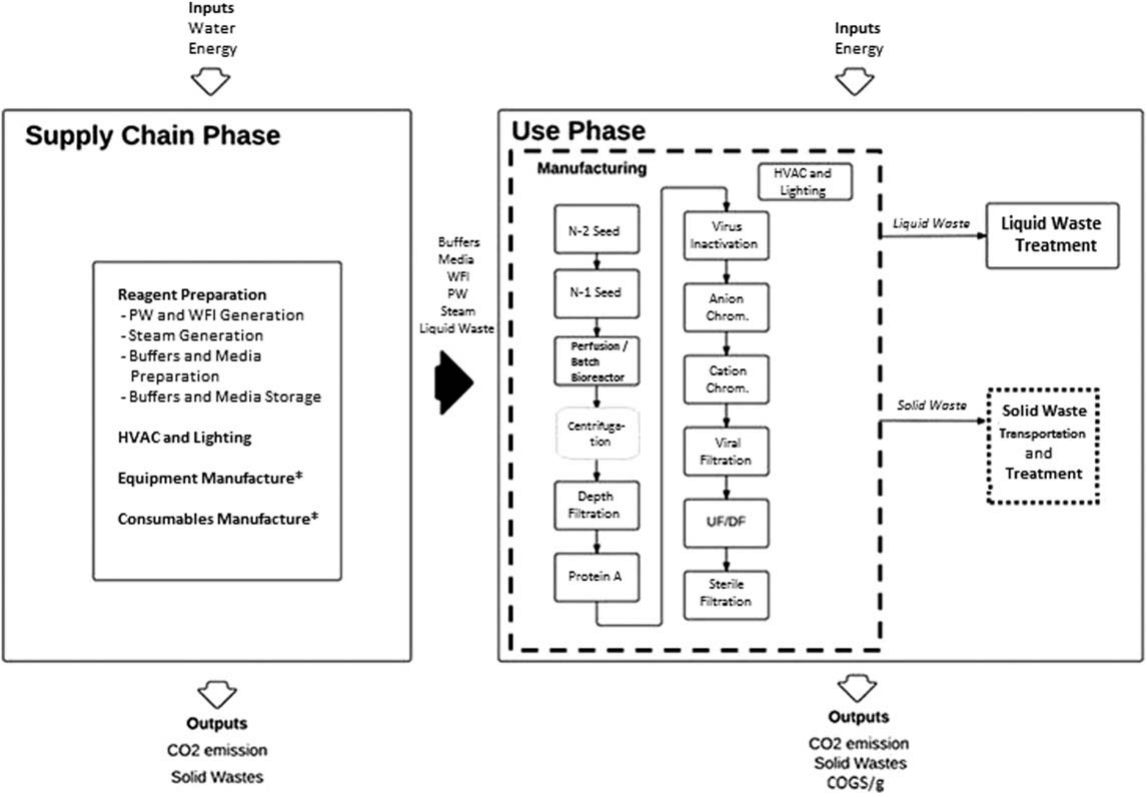
Summary of the cradle‐to‐gate system boundary based on the perfusion process. The operations included a section of the supply‐chain phase and the use phase. The centrifugation step, highlighted in the gray box, was considered in a batch‐based process but not in the perfusion‐based process. BioSolve simulated the manufacturing process (indicated by the dashed line in the use phase), and GABI computed the solid waste transportation and treatment (indicated by the dotted line in the use phase). The energy requirements of the supply‐chain phase were calculated using BioSolve and a facility area classification approach. Components indicated by an asterisk (*) were excluded from the study.

The LCA system boundary of the cradle‐to‐gate approach adopted in this study encompasses the *supply‐chain phase* and the *use phase*, and is summarized in Figure 1. Typically, the supply‐chain phase contains three primary processes: equipment fabrication, consumables manufacture, and reagent preparation. The first two processes are relevant when comparing single versus multi‐use bioprocess systems as consumable components in the manufacturing process can have a significant effect on the overall environmental impact. However, since this LCA study focuses on systems that use stainless‐steel equipment with little solid waste generation upfront, it is sufficient to account only for the environmental impact caused by the reagent preparation process (hence equipment and consumable manufacture was excluded from this study as indicated in Figure 1). The use phase contains the individual unit operations within a mAb manufacturing process, and the management of solid and liquid wastes.^1^

### FB versus perfusion‐based mAb manufacturing processes

There are fundamental differences in the working principles of the two manufacturing processes considered in this study, FB and perfusion‐based processes, driven by the bioreactor type. In a FB bioreactor, the product remains in the bioreactor until the end of the run, while a perfusion bioreactor operates continuously by feeding and withdrawing the culture media while retaining cells within the bioreactor. The pool duration specifies the number of days for which harvest is collected before it is sent for further processing. A typical perfusion bioreactor can operate up to 60+ days before the run is terminated due to, for example, filter clogging, reduction of viable cells, and culture age.^27^ The size of a perfusion bioreactor can reach up to 2,000 L with scalability being limited by the robustness of the cell‐retention system. Generally, a perfusion bioreactor operates at a lower mAb titer than a FB bioreactor. However, a perfusion bioreactor has the ability to process unstable products and cultivate cells at a cell density that is 100 times higher than achieved with a FB bioreactor, allowing for the use of smaller bioreactors. Although, currently, perfusion mode is still perceived as complex and difficult to operate,^28^ recent technological advances in the bioprocessing sector, related particularly to single‐use bioreactors and their linkage with novel cell‐retention systems, are expected to increase the operability and popularity of perfusion‐based bioreactors.^29^, ^30^, ^31^

### Constructing the mass balance model

The study assumed the production of a functional unit of 28 kg‐mAb per year. This was calculated based on the amount of Avastin required for a 5% market penetration to treat lung cancer in the United Kingdom (please refer to Appendix A for a justification and calculation of this production output). To generalize our LCA study, the impact of production scale‐up to outputs greater than 28 kg‐mAb per year was investigated too.

Details related to mAb manufacture were obtained from literature and process simulations using BioSolve, an industry standard bioprocess model from BioPharm Services (Chesham, United Kingdom). The simulation results provided facility data, such as equipment sizing, number of media and buffer containers, Cost of Goods per gram (COGS/g) of mAb, the equipment floor area, and the consumption of PW) and WFI. This data constitutes a mass balance model, which was the foundation for calculating system parameters and quantifying different environmental impacts. Table 1 summarizes the key assumptions used in the bioprocess simulation (the remaining process parameter settings as used by BioSolve are provided in Appendix B).

**Table 1:** Overview of Process Details Assumed for a FB and Perfusion‐Based mAb Manufacturing Process

Technical Properties	FB Process	Perfusion Process
Volume (L)	375	47
Titer (g‐mAb/L)	5	2
Number of production bioreactor runs per year	21	9
Production bioreactor duration (days)	12	30
Pool duration (days)	–	4
Lag phase (days)	–	5
Perfusion rate (VVD)	–	2
Overall downstream process yield	72%	76%
Number of batches (or downstream runs) per year	21	53

*Note*. The properties pool duration, lag phase, and perfusion rate are relevant to the perfusion process only.

To meet a production target of 28 kg per annum we have assumed typical titers for a FB (5g/L) and perfusion process (2g/L) achieved in a manufacturing setup. The lag phase and production bioreactor duration are set to standard values of 5 days and 12 days (FB)/30 days (perfusion), respectively.^32^ Typical pool durations can range from 2 to 7 days,^28^ and, in general, the pool duration is chosen based on protein stability and downstream capabilities. This study has chosen a commonly reported duration of 4 days as the base case. For industrial cell lines, perfusion rates of 0.5–2 VVD (volume‐of‐fresh‐medium/working‐reactor‐volume/day) have been reported.^33^ Volume and number of bioreactor runs per year were computed by BioSolve based on other provided parameters. Note that the perfusion‐based process has a slightly higher DSP yield than the FB‐based process as it does not involve a centrifugation step, and also yields more than double the number of batches (as the pooling strategy adopted leads to more frequent downstream runs). We are aware that manufacturing processes can vary significantly from the base case defined in Table 1. Thus, to investigate if our conclusions are valid for a wider range of processes and scales, we will carry out a sensitivity analysis on the process parameters and production scale‐up study. Ultimately, the insights gained allow trade‐offs between the two process types to be identified and opportunities for process improvements pinpointed.

We want to point out that although BioSolve was used to calculate the annual water consumption of the manufacturing facility, we believe that the consumption of a perfusion process was overestimated because BioSolve assumed the upstream stage was cleaned as frequently as the downstream stages. Realistically, the seed and production bioreactors are cleaned once every perfusion run and not after every purification run. Consequently, while the upstream water consumption was computed using BioSolve, the downstream water consumption was calculated by multiplying the water consumption of a single downstream run (which was computed by BioSolve) by the number of downstream runs per run.

### Constructing the energy balance model

A detailed energy balance scheme was beyond the capabilities of BioSolve. Hence, we developed our own model for this study based on literature data and vendor discussions (Appendix C provides the key assumptions for constructing the energy balance model). BioSolve provided crucial inputs to the energy balance model such as water usage, production runtime, and facility floor area. These parameters were linked with our energy model capture energy usage related to manufacturing operations, HVAC, water production, reagent preparation, lighting, and waste management.

The energy required to operate each unit operation in the mAb manufacturing process was calculated by multiplying the equipment power input by the operating duration per year, while the energy to operate the HVAC system was calculated based on the floor area (as done in similar form in Ref. 21). BioSolve provided both the total floor area for each area classification and the equipment operating duration per year. Table 2 shows the average energy consumption for each area classification. The grades refer to different clean room types as required for different tasks in the manufacturing process (please refer to Refs 21, 34 for a detailed explanation of the grades).

**Table 2:** HVAC Energy Consumption for Each Class of Facility Space

Area Classification (grade)	Average Energy Consumption per Floor Area (kWh/m^2^)
B	854
C	237
D	119
U	47.0

### Evaluating solid waste management

This study investigated the environmental impact of three solid waste treatment options: landfill, incineration, and pyrolysis. The analysis of landfill and incineration was performed using the GaBi software package^35^ with the GaBi bundled professional database^36^ serving as the principal data source. The GaBi software package is an established LCA modeling software to quantify environmental impacts and has been used widely in various industries, such as wine production,^37^ wood panel industry,^38^ and power systems,^39^ to name a few. Its application in the biopharma industry is still rare (see e.g., Refs 19, 20, 21) but is expected to increase in popularity significantly as the industry matures. Plastics represent the major part of the solid waste generated by the manufacture of mAb, and provide the driving multiplying factors in the estimation of water consumption, energy requirements, and CO_2_ emission levels of the landfill and incineration processes. The GaBi database does not contain data for the third treatment option, pyrolysis. Discussions with industrial partners, including Royal Dahlman (http://www.royaldahlman.com/), allowed us to make reasonable assumptions about the performance of pyrolysis. Table 3 summarizes the multiplying factors for all three waste treatment options.^2^

**Table 3:** Environmental Multiplying Factors of Three Solid Waste Treatment Options, Landfill, Incineration, and Pyrolysis, Commonly Used in the Biopharmaceutical Industry

Environmental Impact Type	Landfill	Incineration	Pyrolysis
Water consumption factor (kg‐water/kg‐waste)	40	7	7
Energy consumption factor (MJ/kg‐waste)	1	−7	−3
CO_2_‐eq emission (kg‐CO_2_eq/kg‐waste)	1	2	1

## Results and Discussion

### Which process is generally more environmentally‐friendly and/or economical?

Table 4 summarizes the crucial environmental impact metrics—water consumption, solid waste generation, energy consumption, and CO_2_ emission levels—and COGS/g for a FB and perfusion‐based process based on a functional unit of 28 kg‐mAb production per year. From Table 4 it is apparent that a FB process is significantly more environmentally‐friendly than a perfusion‐based process, while having only slightly higher COGS/g. Note, the level of CO_2_ emission is a function of the energy requirement (the interested reader is referred to Ref. 40 for more details on this relationship) causing both metrics to be proportional to each other. The results are also in alignment with those reported by Pollock et al.,^3^ though that study did not evaluate levels of energy consumption and CO_2_ emissions.

**Table 4:** Environmental Impact Metrics and COGS/g for a FB and Perfusion‐Based Process Based on 28 kg/Year of mAb

Process Performance Metric	FB	Perfusion	Relative Difference Between FB and Perfusion
Water consumption (kg/year) × 10^6^	1.1	1.5	35.1%
Solid waste (kg/year) × 10^3^	0.7	1.3	93.1%
Energy requirement (MJ/year) × 10^6^	1.3	1.5	16.7%
CO_2_ emission (kg/year) × 10^6^	0.17	0.20	17.4%
COGS/g (USD/g)	494	504	2.0%

To put the results into context with the UK domestic consumption,^41^, ^42^, ^43^ a perfusion‐based process consumed per year an equal amount of water as 28 people, required an equivalent amount of energy as around 100 households, and generated a little more solid waste than a single person would. The following sections analyze the individual environmental impact categories in more detail.

### What are the key drivers for water consumption?

**Figure 2:**
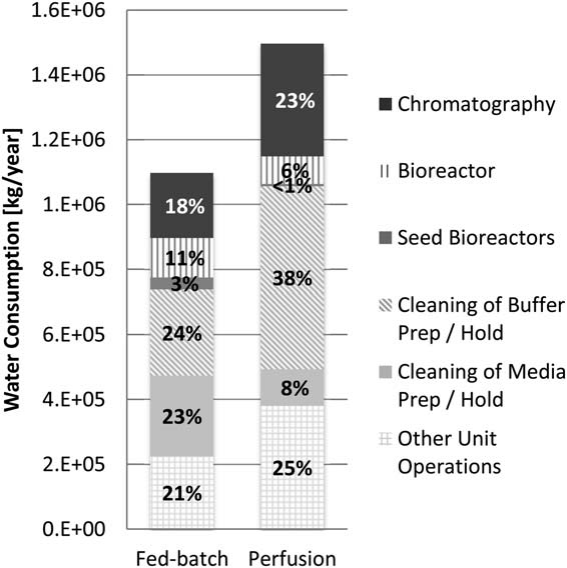
Comparison of the overall water consumption of a FB and perfusion‐based process. Percent contributions are shown for different process activities including chromatography (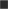), production bioreactor (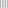), seed bioreactors (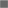), cleaning of buffer preparation / hold (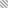), cleaning of media preparation / hold (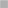), and other unit operations (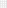). For both processes, the supply‐chain and use phase comprised 46% and 54% of the total water consumption, respectively. Basis for both cases is 28 kg‐mAb/year.

Water consumption varied across the upstream and downstream stages of a manufacturing process as can also be observed from Figure 2. Interestingly, while the FB‐based process consumes less water overall, for upstream activities, it consumes around double the amount of water than a perfusion‐based process (204 versus 407 tonnes of water/year). This result may seem non‐intuitive because the perfusion bioreactor requires a constant input of fresh media and thus one could expect the consumption for upstream activities to be higher. The explanation for this observation is that a FB‐based process undergoes cleaning after each batch. In the presence of many batches (e.g., during commercial manufacture stage or blockbuster drug) this turns out to consume more water than maintaining a continuous perfusion‐based process that needs occasional cleaning only (21 cleans for FB versus 9 cleans for perfusion, as can be seen in Table 1). While the perfusion‐based process consumes less water for upstream activities, the water demand is almost double for downstream activities (1290 versus 690 tonnes of water/year). This is due to the fact that the perfusion‐based process operates its downstream process more than twice as frequently compared with a FB process leading to significantly higher CIP and SIP costs, which make up more than 85% of the total water consumption.

### What are the key drivers for energy requirements?

**Figure 3:**
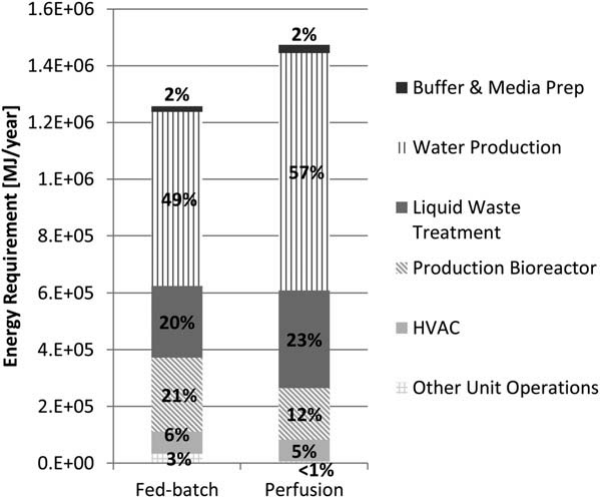
Comparison of the total energy requirement of FB and perfusion processes. Percent contributions are shown for different process activities including buffer & media preparation (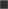), water production (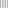), liquid waste treatment (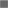), production bioreactor (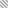), HVAC (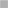), and other unit operations (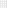). For the FB process, the supply‐chain and use phase comprised 45% and 55% of the energy requirement, respectively. For the perfusion‐based process, the supply‐chain and use phase comprised 37% and 63% of the energy requirement, respectively. Basis for both cases is 28 kg‐mAb/year.

Figure 3 shows the energy usage of FB and perfusion‐based processes for different manufacturing activities. It can be seen that water production, which includes the production of PW and WFI, and liquid waste treatment are the most energy intensive steps, accounting for approximately 50% and 20% of the overall energy demand, respectively. This means there is a positive correlation between water and energy usage, which is also reflected in Table 4 with the FB‐based process being more economical in terms of both energy and water usage.

Figure 3 highlights also that the production bioreactor is the most energy intensive unit operation accounting for approximately 90% of the total energy requirement among all the unit operations. This is due to the long running time of a bioreactor and the complex setup investing energy in temperature control, agitation, and gas sparging. By comparison, a modest amount of energy is invested in fluid transport and mixing in other unit operations. The FB process consumed more energy in its “other unit operations” due to the need for large seed bioreactors.

It is worth noting that the HVAC system contributed around 5% to the overall energy demand but that this contribution could have been significantly greater if the energy‐balance model accounted for geographical‐dependent factors too (e.g., temperature control). HVAC energy requirements of up to 4,000 kWh/m^2^ (which is more than 10‐fold higher than the consumption reported in this study)^44^ and contributions of up to 50% to the overall energy demand^14^ have been reported in the literature. However, since the energy requirements for the HVAC systems for a FB and perfusion‐based process differed by only 1% and did not depend heavily on the process being used (but rather on the geographical factors), a larger energy requirement of the HVAC systems is not expected to impact the ranking of the two processes in terms of energy and water usage.

### What are the key drivers for solid waste generation?

**Figure 4:**
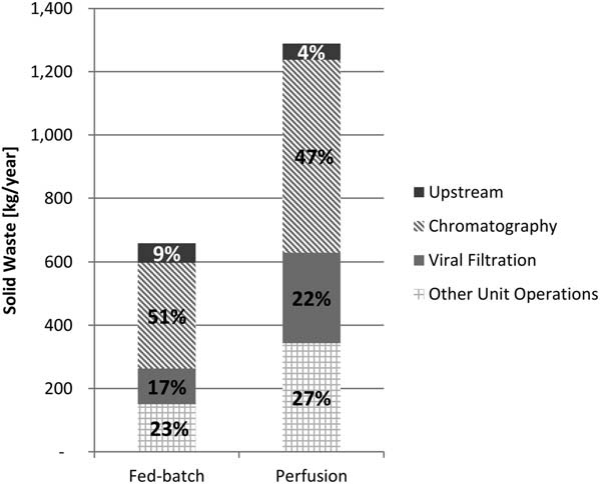
Comparison of the total solid waste generated by a FB and perfusion process. Percent contributions are shown for key process activities including upstream (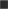), chromatography (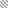), viral filtration (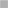), and other unit operations (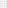). Waste was generated by the supply‐chain phase only. Basis for both cases is 28 kg‐mAb/year.

Figure 4 shows the key unit operations contributing to solid waste generation for a FB and perfusion‐based process. It can be seen that the composition of solid wastes were similar for both manufacturing processes with the top three components being attributed to: (i) the three chromatography steps (≈50% of total waste with 6–24% being resin waste and 76–94% filters), (ii) viral filtration (≈20% of total waste), and (iii) upstream activities (<10% of total waste). Consequently, since a perfusion‐based process performs more (chromatographic) downstream runs per year than a FB‐based process, it generated also more downstream‐related waste (1.2 versus 0.6 tonnes). On the other hand, a perfusion‐based process is more economical on the upstream side (≈20% less waste than a FB‐based process) as it requires fewer upstream runs.

**Figure 5:**
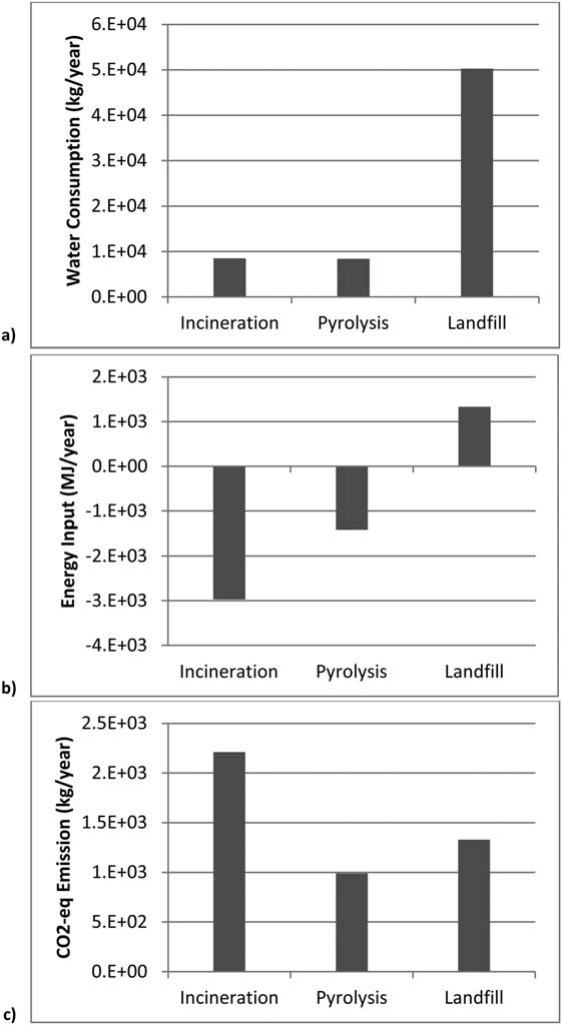
Impact of each waste treatment option on key environmental metrics (a) water consumption, (b) energy demand, (and c) CO_2_ emission. Basis in all cases is 28 kg‐mAb/year.

Figure 5 shows the water consumption (Figure 5a), energy requirement (Figure 5b), and CO_2_ emission (Figure 5c) associated with the three waste treatment options, incineration, pyrolysis, and landfill. The options of incineration and pyrolysis consumed five‐times less water than landfill, and were able to generate net energy that could be recycled back to supply the manufacturing process (hence the negative energy input).^3^ Incineration generated most energy but it also emitted most CO_2_ because of the combustion process involved in this waste treatment option. In summary, pyrolysis seems to strike the best balance between environmental impact and the potential to recycle energy, while landfill performs poorly in terms of both aspects.

However, in contrast to the environmental impact generated by the manufacturing process per se, waste treatment represents only a tiny fraction (maximum 3% across the two processes, FB versus perfusion, and impact categories). The impact is also rather little compared with domestic usage: for example, the water consumption of the landfill option is similar to the annual water usage of a single person, and the CO_2_ emission associated with the incineration option is less than the annual amount emitted by a single passenger vehicle.^45^

### What are the key drivers for COGS/g?

Although the COGS/g of the FB and perfusion‐based process were similar ($494 versus $504 as can be seen from Table 4), there is a difference in the cost breakdown: The perfusion‐based process has a lower capital cost (57% versus 48%) due to smaller pieces of equipment being deployed, especially a smaller production bioreactor. However, this comes at the expense of higher material, consumable, and labor costs since the perfusion‐based process employs more downstream runs per year. In other words, a perfusion‐based process is associated with a lower capital investment but higher variable costs, which is a setup suitable for manufacturing drugs with uncertain demand forecast as might be the case during clinical trials.

### How sensitive is the environmental impact and COGS/g to process parameter changes?

The results above were obtained using a fixed set of process parameters. However, in reality, the same parameters may be subjected to batch‐to‐batch variation, such as titer, and it may also be possible to restructure parts of a process (e.g., in a facility fit scenario) or even design a new process (e.g., in a new facility design scenario). A sensitivity analysis can help identifying parameters that need careful consideration in such scenarios so as to deal better with uncertainty, be it of environmental or economical nature.

**Figure 6:**
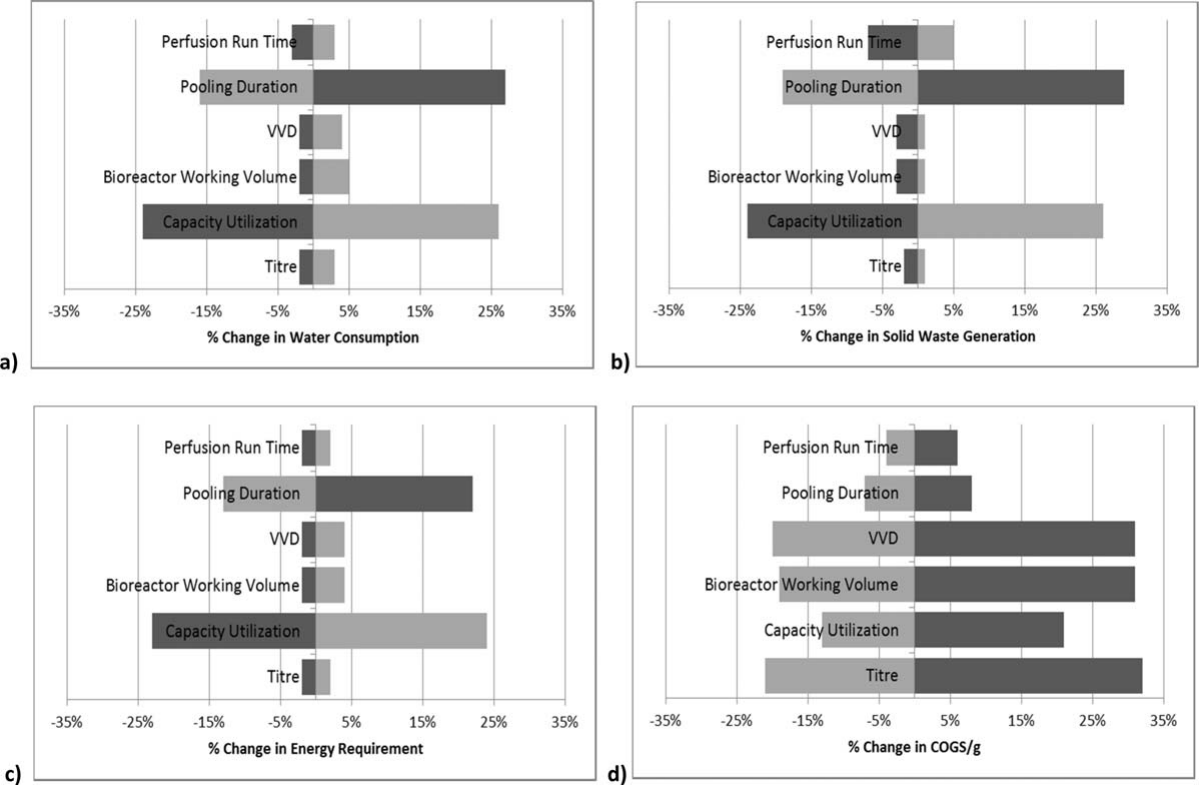
Results of sensitivity analysis showing impact of several process variables—perfusion run time, pooling duration, VVD, bioreactor working volume, capacity utilization, and titer—for a perfusion‐based process on several environmental metrics—(a) water consumption, (b) level of solid waste generation, (c) energy demand—, and (d) COGS/g. The process variables are varied one at the time by −25% (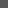) and +25% (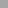). Basis in all cases is 28 kg‐mAb/year.

Figure 6 shows the influence of changes in crucial process parameters—including titer, capacity utilization,^4^ bioreactor working volume, perfusion rate (VVD), pooling duration, and perfusion run time—on water consumption (Figure 6a), solid waste generation (Figure 6b), energy requirements (Figure 6c), and COGS/g (Figure 6d) for a perfusion‐based process. In general (except for COGS/g), it can be seen from the figure that the capacity utilization and pooling duration are the most influential variables with changes of ±25% in either variable leading also to a change of around ±25% on the output of three impact categories. Varying the other process variables by some degree leads to a change of around 5% in the different impact categories. On the other hand, COGS/g is most sensitive to variations in the titer, bioreactor working volume, and VVD.^5^ Note that while increasing VVD results in an increased usage of media and thus increases the material and consumable costs, the bioprocess model assumes that a higher VVD increases also the (annual) throughput. In this particular setting, relatively speaking, the throughput increased more significantly than the costs leading to lower COGS/g.

**Figure 7:**
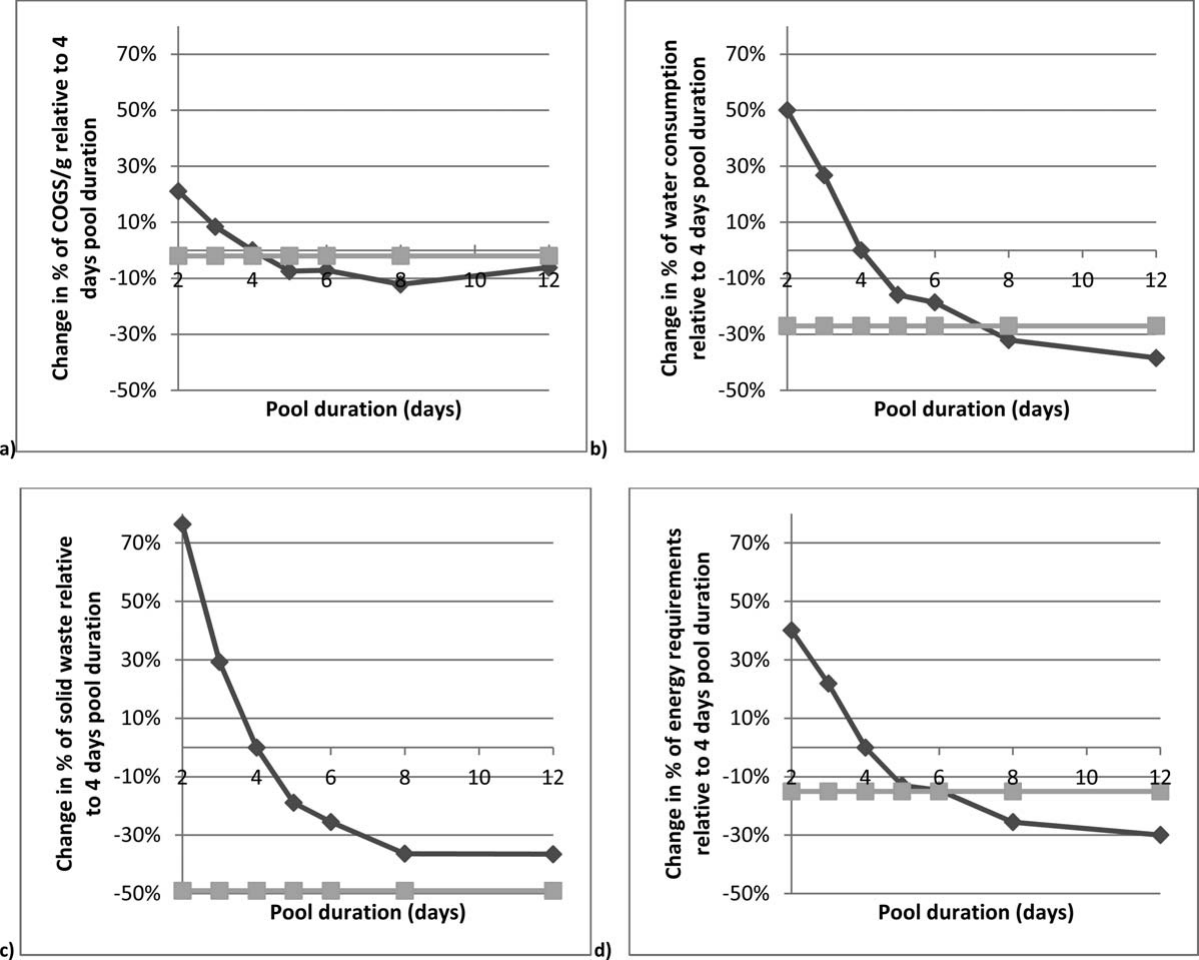
Analysis of the impact of pool duration on (a) COGS, (b) water consumption, (c) solid waste generation, and (d) energy requirements for a perfusion‐based process (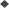). The impact is measured relative to a perfusion process with a pool duration of 4 days (hence the 0% change in metrics at pool durations of 4 days). As a reference, each plot shows the output of the batch‐based process (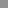 ) (the straight horizontal line), which is independent of the pool duration and hence constant.

Note that the pooling duration is defined as the number of days for which harvest is collected before further processing and thus affects both the upstream and downstream setup. For example, keeping the total production amount constant, a longer pool duration (i.e., fewer upstream runs) leads to less frequent purification runs and a larger downstream batch. The sensitivity analysis shows that an increase in pool duration has a positive effect on both the COGS/g and the environment. In fact, from Figure 7 it can be seen that a pooling duration between 5 and 8 days and longer allow the perfusion‐based process to be more economical (achieved for pooling durations of 5 or more days) and environmentally‐friendly (achieved for pooling duration of 6 and more days) than the FB process (except for solid waste generation as indicated in Figure 7c). Of course, if a longer pool duration can be implemented depends strongly on the stability of the protein and the downstream capabilities (e.g., equipment and vessel size) of the process.

Finally, it is worth pointing out that water consumption was more dependent on the frequency of cleaning operations than the size of downstream equipment. Put simply, a downstream process with larger equipment but lower frequency of cleaning operations consumed less water.

### How sensitive is the environmental impact and COGS/g to changes in the production scale?

**Figure 8:**
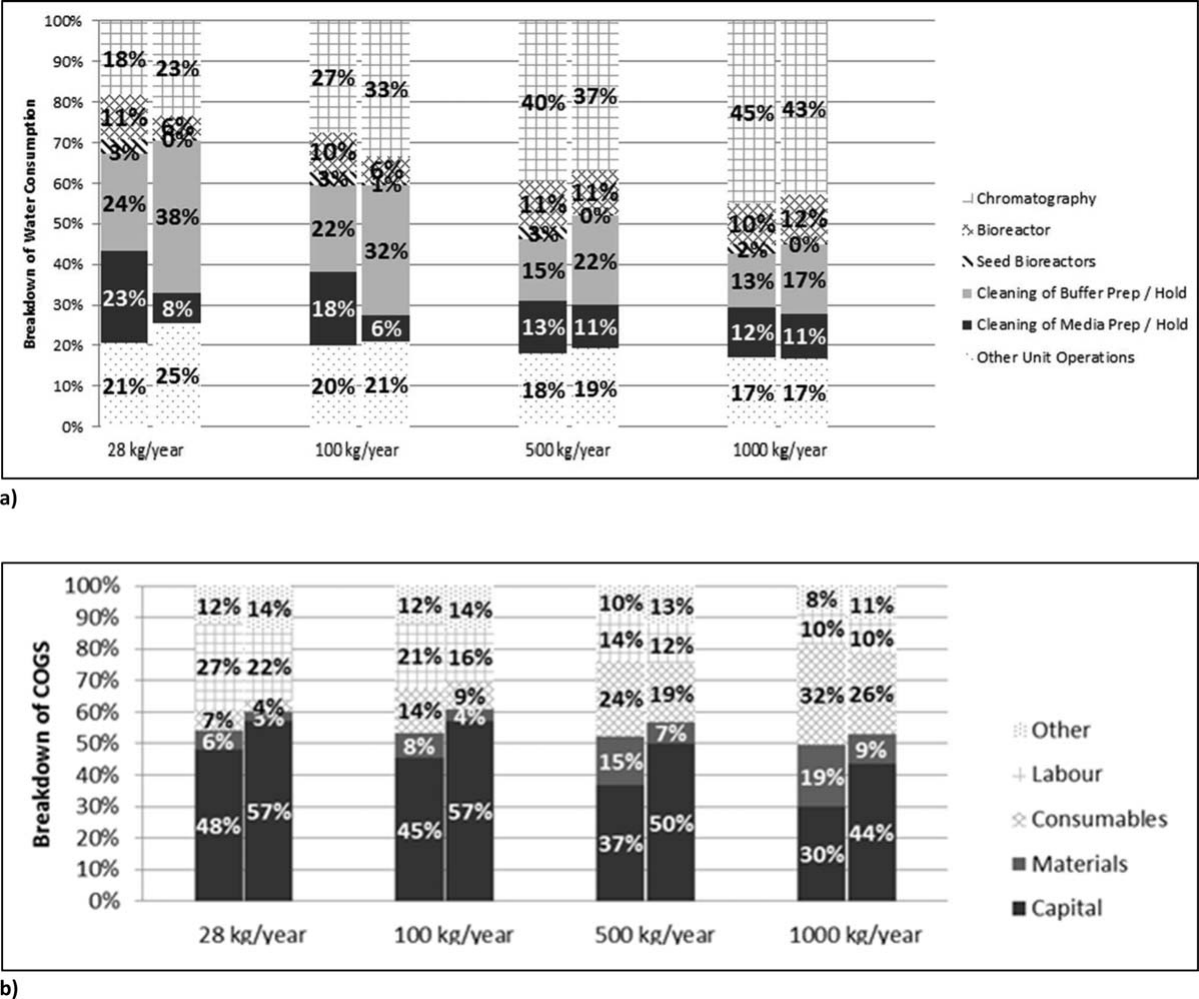
Comparison of the (a) relative water consumption and (b) COGS breakdown of a FB (left bar for each scale) and perfusion‐based process (right bar for each scale) for production scales ranging from 28 to 1000 kg/year.

The framework developed can be used to assess the environmental impact and COGS/g of different production scales. As an example, Figure 8 highlights the impact on (relative) water consumption (Figure 8a) and COGS/g (Figure 8b) for a FB and perfusion‐based process for production scales ranging from 28 to 1000 kg‐mAb/year. It can be seen from Figure 8a that the distribution of the relative water consumption changes significantly with the scale (the change was most significant amongst the three impact categories considered, water consumption, energy usage, solid waste generation). In particular, the relative water consumption associated with the chromatography units increased with the scale, while the water consumption required for cleaning of buffer preparation/holding tanks decreased significantly. The former was due to a rapid increase in the amount of buffer solutions required to run a larger chromatography column (needed for large production scales). The latter is due to the fact that the increase in buffer solutions dominated the impact on water consumption induced by larger equipment (tanks) needed for buffer preparation/holding. When comparing the total water consumption between the two processes for the different scales, the perfusion process remained the more water‐demanding process consuming around 25% more than the FB process (as observed previously in Table 4 and Figure 2 for a scale of 28 kg‐mAb/year).^6^

Figure 8b demonstrates the impact of scale on the COGS/g breakdown. It can be seen that the capital contribution is becoming significantly lower as the scale increases, while the contribution of materials and consumables increases. This observation can be attributed to the economy of scales.

## Conclusion

Economic factors are currently used to compare manufacturing strategies in the biopharmaceutical industry. While new developments are pushing this industry toward greener manufacturing processes, only a very few environmental studies have been conducted on the manufacture of biopharmaceuticals. This study studied the use of a simulation tool to assist in performing an LCA study. The use of a commercial software tool, BioSolve, enabled this study to be completed within a reasonable timeframe. The ability to evaluate quickly the environmental impacts from the simulation results allows a decision‐maker to weigh economic and environmental factors simultaneously at the process design stage.

Under the assumptions used and based on a 28 kg/year output of a mAb, the perfusion‐based process generated larger environmental impacts compared with the FB process due to more frequent cleaning of the downstream stage when the pool duration was 4 days. However, both processes had similar water and energy consumption levels when the pool duration was increased to 8 days. The study also found that water consumption greatly affected energy requirements as energy was used in water production and liquid waste treatment. CIP and SIP systems were identified as steps with major environmental impacts.

Since water consumption influences energy requirements and CO_2_ emission levels, and a significant proportion of water was consumed in cleaning operations, the shift to single‐use equipment and a fully continuous mAb process may be expected to reduce the environmental impacts.

The environmental impacts from three waste management scenarios were evaluated. Arguably, the incineration process was the most desirable option as it converted waste effectively into energy, but at the expense of emitting more CO_2_. The solid wastes generated comprised mostly non‐degradable plastics which are not ideal for landfill.

The study revealed also the impact on the economics of a process is different from the environmental impact. For example, while increasing the facility capacity increases water and energy consumption, it can have a positive impact on the cost of goods due to economics of scale.

Future studies could focus on comparing single‐use with traditional bioprocess systems. Here, the system boundary must be expanded to examine the whole life‐cycle which also includes equipment fabrication and consumables manufacture in the supply‐chain phase and equipment disposal in the end‐of‐life phase. Further studies could include other environmental impact criteria of interests such as acidification, global warming potential and aquatic toxicity. Finally, in addition to quantifying the direct environmental impact of a plant as done in this study, it would also be interesting to investigate the indirect impact of a plant, which would depend on factors such as the location of the plant, local regulations and logistics.

## Footnotes

1 Note, a perfusion‐based process does not require a centrifugation step during the clarification stage since cells are retained in the perfusion bioreactor.

2 The input data to GaBi consists of the environmental multiplying factors as specified in Table 3, and the average distance from the manufacturing plant to the waste yard, which was set to 25 km, a value we obtained from discussions with industrial partners.

3 In principal, the landfill process could generate energy too by collecting methane gas obtained as a by‐product from anaerobic digestion. However, since non‐biodegradable plastics do not readily decompose, they do not generate methane gas.

4 The capacity utilization is used to set the number of days in a year a facility is run for, i.e., #operating days = capacity utilization × 365. In the base case, the capacity utilization is set to 0.8 (or 80%).

5 In the case of a FB‐based process, the parameters VVD, pooling duration, and perfusion run time can be neglected. The impact of the other three variables, titre, capacity utilization, and the bioreactor working volume, on the environment and the COG is very similar to impact observed for the perfusion‐based process.

6 The contribution of the centrifugation step (which was used by the FB‐based process only) to the water consumption was around 5% at each scale. A similar contribution was observed for the energy requirement.

## Appendix A Functional Unit Calculation

***The following assumptions were made***:

1. The monoclonal antibody is Avastin.
2. In 2011, there were 141,000 patients with lung cancer in the United Kingdom.^52^
3. Only 80–85% of lung cancer patients have non‐small cell lung cancer.^53^
4. Approximately 75% of patients of non‐small cell lung cancer have non‐squamous non‐small cell lung cancer.
5. Only 40 % of non‐squamous non‐small cell lung cancer patients are in the stage that can be treated by Avastin.
6. Five percent market penetration capacity of Avastin for treating lung cancer patients in the United Kingdom.
7. Assume a dose size of 15.5 g per person per year.

*Amount of mAb required* = 141,000 × 0.85 × 0.75 × 0.4 × 0.05 × 15.5 = 27,865 mg per year ≈ 28 kg per year.

## Appendix B Parameters for a FB and Perfusion‐Based Manufacturing Process

**Table:**

Unit Operation Parameter	Setting	Unit Operation Parameter	Setting
***Seed bioreactors***		***Virus inactivation***	
Batch split ratio	1	Operational yield	100%
Batch pooling ratio	1	Base vol	0.50%
***FB bioreactor***		Acid vol	1.50%
Titer (g/mL)	2	***Anion chromatography***	
Working volume	45	Operational yield	89%
Operational yield	100%	Capacity (g/L)	30
Feed ratio in	10	Bed height (cm)	20
***Perfusion***		Product CVs	3
Titer (g/mL)	2	Target cycles	1
Working volume	45	Max # reuses	50
Operational yield	100%	***Cation chromatography***	
Perfusion rate (VVD)	2	Operational Yield	98%
Total run time (days)	60	Capacity (g/L)	30
Initial growth phase (days)	5	Bed height (cm)	20
# Days media prep	1	Product CVs	3
***Centrifugation***		Target cycles	1
Operational yield	95%	Max # reuses	50
Centrate	85%	***Viral filtration***	
Duration (h)	4	Operational yield	98%
***Depth Filtration***		Flux (LMH)	100
Operational yield	95%	Duration (h)	4
Flux (LMH)	2	***Ultrafiltration/diafiltration***	
Duration (h)	4	Operational Yield	98%
***Protein A chromatography***		Flux (LMH)	50
Operational yield	97%	Duration (h)	4
Capacity (g/L)	30	Concentration factor	10
Bed height (cm)	20	Diavolumes	8
Product CVs	3	Max # reuses	10
Target cycles	4	***Sterile filtration***	
Max # reuses	200	Operational yield	98%
		Flux (LMH)	200
		Duration (h)	2

## Appendix C Basis of Calculation for Energy Balance

**Table C1:** Energy consumption of the production facility

Item	Energy Required	Source/Comment
PW and CIP production	558 (kJ/L)	Ref. 46
SIP production	797 (J/s‐L)	Energy to heat water from 25°C to 250°C
Filtration	50 (J/s‐m^2^)	Ref. 47
Media and buffer preparation	69.0 (J/s‐L)	Ref. 48
Liquid waste treatment (heat to 80°C)	639 (J/s‐kg‐water)	Inactivation of mammalian cells at 85°C^49^
Lighting	15 (J/s‐m^2^)	Ref. 50

**Table C2:** Energy consumption of the unit operations

Item	Working Volume	Energy (J/s)	Equipment Example
Bioreactor	50 mL to 5 L	126	WAVE Bioreactor 2/10
	100 mL to 25 L	630	WAVE Bioreactor System 20/50
	25 L	900	ReadyToProcess WAVE 25
	10 L to 100 L	7200	WAVE Bioreactor™ 200 system
	500 L	12000	WAVE Bioreactor System 500/1000
Ion chromatography	1.5 mL/min	240	Cecil IonQuest Ion Chromatography System
	7.5–510 L/h	600	ÄKTA ready
	up to 2000 L/h	1700	Bio‐Rad InPlace Columns Product Information
